# Risk factors of neuropathic pain in multiple sclerosis: a retrospective case-cohort study

**DOI:** 10.3389/fimmu.2024.1309583

**Published:** 2024-01-29

**Authors:** Huiying Ouyang, Xiaojun Li, Haoyou Xu, Yibo Zhan, Zequan Zheng, Guixian Chen, Zhenzhen Lou, Haoxuan Chen, Jiahui Zhang, Hui Mao, Changlin Zhang, Lulu Qin, Yuanqi Zhao, Min Zhao

**Affiliations:** ^1^ The Second Clinical College of Guangzhou University of Chinese Medicine, Guangzhou, China; ^2^ State Key Laboratory of Dampness Syndrome of Chinese Medicine, The Second Affiliated Hospital of Guangzhou University of Chinese Medicine, Guangzhou, China; ^3^ Department of Neurology, The Second Affiliated Hospital of Guangzhou University of Chinese Medicine, Guangdong Provincial Hospital of Chinese Medicine, Guangzhou, China

**Keywords:** spinal cord lesions, basal ganglia, multiple sclerosis, neuropathic pain, MRI

## Abstract

**Background:**

Pain is a common symptom in multiple sclerosis (MS), especially neuropathic pain, which has a significant impact on patients’ mental and physical health and quality of life. However, risk factors that related to neuropathic pain, still remain unclear.

**Objective:**

The study aimed to explore the risk factors of neuropathic pain among MS patients.

**Materials and methods:**

This retrospective study examined the consecutive patients diagnosed with MS in the Department of Neurology of Guangdong Provincial Hospital of Chinese Medicine between August 2011 and October 2022. Neuropathic pain was defined as “pain arising as a direct consequence of a lesion or disease affecting the somatosensory system”. Demographic and clinical features were obtained from the electronic system of the hospital.

**Results:**

Our cohort revealed that the prevalence of patients with neuropathic pain in MS was 34.1%. The results indicated that the longer the spinal lesions, the greater the neuropathic pain risks (2-4: OR, 13.3(2.1-82), >5: OR, 15.2(2.7-86.8), p for tread: 0.037). Meanwhile, multivariate regression analysis showed that cervical and thoracic lesions (OR 4.276, 95% CI 1.366-13.382, P = 0.013), upper thoracic lesions (T1-T6) (OR 3.047, 95% CI 1.018-9.124, P = 0.046) were positively correlated with neuropathic pain, while basal ganglia lesions (OR 0.188, 95% CI 0.044-0.809, P = 0.025) were negatively correlated with neuropathic pain among MS patients.

**Conclusion:**

Extended spinal lesions (≥3 spinal lesions), cervical and thoracic lesions, upper thoracic lesions were independent risk factors of neuropathic pain among MS patients. Furthermore, our study found that the longer the spinal lesions, the greater the neuropathic pain risks.

## 1 Introduction

Multiple sclerosis (MS) is an autoimmune, inflammatory disorder of the central nervous system characterized by multiple demyelination scattered in the brain and spinal cord with a chronic course and various clinical symptoms. Pain is one of the most troubling and refractory clinical symptoms in MS(1–3), especially the neuropathic pain(4, 5). The prevalence of patients with neuropathic pain in MS varies between 21% and 58%(6–8), which interferes the quality of life domains(3, 5). There are several definitions of neuropathic pain, such as International Association for the Study of Pain (IASP) criteria(9), Networks-American Pain Society Pain Taxonomy (AAPT) criteria(10), and some validated questionnaires(11). The IASP definition of neuropathic pain was that “pain arising as a direct consequence of a lesion or disease affecting the somatosensory system”, which was used in our study. Meanwhile, a study had divided the neuropathic pain into three types: ongoing extremity pain, trigeminal neuralgia and Lhermitte’s phenomenon(12). So far, both the underlying mechanism and effective treatment of neuropathic pain associated with MS are still unclear(13). According to previous studies, neuropathic pain may arise from lesions within the somatosensory nervous system(7, 14) which was found to be closely related to the pathophysiology of MS(4). Although recent research suggested female, higher disability and longer course were more likely to suffer neuropathic pain (15), several clinical studies suggested that clinical factors (such as disability and disease duration) and demographic features (age) were associated with neuropathic pain in patients with MS(16, 17). Since the risk factors of neuropathic pain are still unclear, our study aims to investigate the risk and protective factors of neuropathic pain among MS patients.

## 2 Methods

### 2.1 Patients

We retrospectively screened the consecutive patients diagnosed with MS in the Department of Neurology of Guangdong Provincial Hospital of Chinese Medicine between August 2011 and October 2022. All patients met the 2017 revised McDonald criteria. Patients with incomplete clinical data were excluded. Neuropathic pain (NP) was defined as “pain arising as a direct consequence of a lesion or disease affecting the somatosensory system” and was assessed by a neurologist according to the IASP criteria (9). It means that “If patient’s pain is described within the area affected by an MS lesion in the brain or spinal cord, and associated with sensory changes in the same neuroanatomically plausible distribution, it is considered that the person has neuropathic pain”(10). Headache (with the exception of trigeminal neuralgia) is not considered as neuropathic pain in our study. Patients were classified into NP Group and non-NP Group based on the presence of neuropathic pain. Then, we analyzed the risk factors of MS patients. Demographic and clinical features such as sex, age of the first episode, Extended disability status Scale (EDSS) of the first onset, symptoms of the first episode including neuropathic pain and pain location, localization of lesions of the whole course of the disease on Magnetic Resonance Imaging (MRI) were collected from the hospital system database. In our study, two associate professors respectively interpreted the imaging data to determine their locations of lesions and the length of involved spinal segments. If the two neurologists had different views, a third senior neurologist made the final judgment.

The study was approved by the Institutional Review Boards (Ethical Committee of Guangdong Provincial Hospital of Chinese Medicine ID ZE2022-165-01). Written informed consents were not required.

Study sample size was based on our previous study, which found that extended spinal lesions was more prevalent among patients with neuropathic pain in NMOSD (OR 4.41, 95% CI 1.54-12.62)(18). We assumed the occurrence rate of NP among MS patients with extended spinal lesions was 65% and OR was 2. Then, a sample size of 80 patients with an alpha of 0.05 and a beta of 0.10 was required to reach the power over 90%.

### 2.2 Statistical analyses

Continuous variables were presented as means, standard deviations (SD) or medians and ranges, while categorical factors were presented as number with percentage. The categorical variables were analyzed with a chi-square test. Using an independent two-sample Student’s t-test and a nonparametric test for continuous variables with normal distribution and data that were not normally distributed, respectively. To assess the independent risk factors of neuropathic pain among MS patients, the association between demographic and clinical variables and neuropathic pain was tested using univariate and multivariate linear regression models. P values < 0.05 were considered statistically significant.

## 3 Result

**Figure 1:**
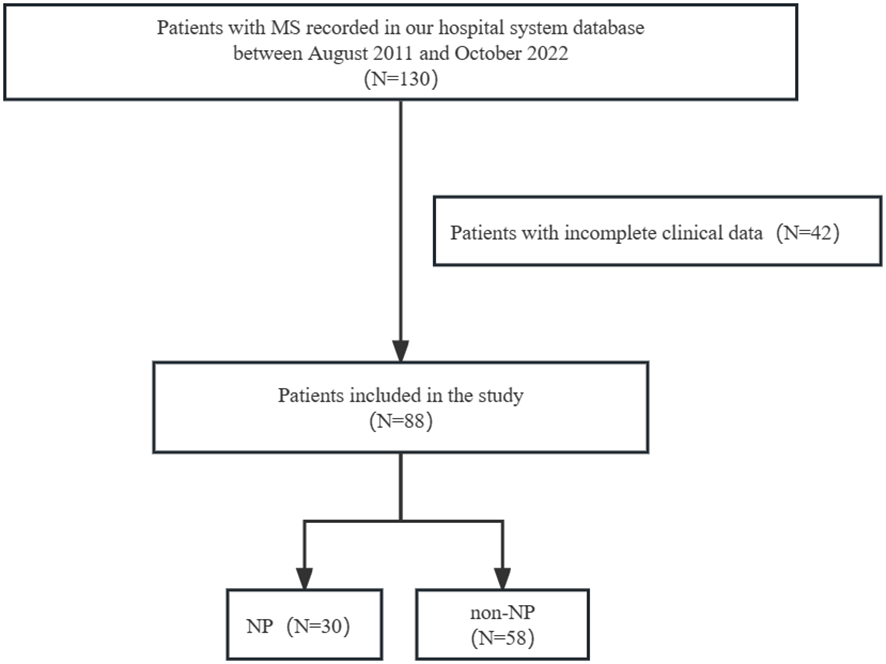
Flow chart.

Totally, 130 patients with MS were screened and 88 patients were enrolled in our study. Patients with incomplete clinical data were excluded (N=42) (**Figure 1**). Out of 88 patients, 62(70.5%) were female, with an average age of 38.3(SD 14.0) years at the first attack and an EDSS score of 3.1(SD 2.1) at the first onset. The most reported pain was observed in lower limb (25.0%), head (20.5%), and upper limb (20.5%) among the total population. Nine patients only had headaches were not included in NP group. Spinal cord lesions were detected in 64 (77.1%) MS patients on MRI. The most common type of spinal lesion was cervical lesions (70.4%) followed by thoracic lesions (64.1%). 47(57.3%) patients had extended spinal lesions (≥3 spinal lesions). 31(37.8%) patients had upper thoracic lesions (T1-T6). 31(37.8%) patients had cervical and thoracic lesions. 29(35.4%) patients had lower thoracic lesions (T7-T12). 24(29.3%) patients had isolated cervical lesions. 17(21.0%) patients had extended thoracic lesions (≥4 thoracic lesions). 8(9.8%) patients had isolated thoracic lesions (**Table 1**).

**Table 1:** Demographic and clinical characteristics of patients with and without neuropathic pain.

Patients	Total (n=88)	NP group (n=30)	Non-NP group (n=58)	P value
Age (years) (mean ± SD)	38.3 ± 14.0	40.1 ± 12.7	37.1 ± 14.6	0.262
Gender				0.575
M (n, %)	26(29.5)	10(33.3)	16(27.6)	
F (n, %)	62(70.5)	20(66.7)	42(72.4)	
EDSS (mean ± SD)	3.1 ± 2.1	3.8 ± 2.7	2.7 ± 1.6	0.055
Patients with brain MRI abnormalities (n, %) *	82(96.5)	28(96.6)	54(96.4)	1.000
Periventricular	53(62.4)	17(58.6)	36(64.3)	0.609
Cortical or subcortical	55(64.7)	17(58.6)	38(67.9)	0.398
Frontal lobe	43(50.6)	11(37.9)	32(57.1)	0.093
Parietal lobe	35(41.2)	9(31.0)	26(46.4)	0.172
Occipital lobe	19(22.4)	6(20.7)	13(23.2)	0.791
Temporal lobe	30(35.3)	6(20.7)	24(42.9)	0.043
Corona radiata	47(55.3)	14(48.3)	33(58.9)	0.349
Basal ganglia	33(38.8)	6(20.7)	27(48.2)	0.014
Half oval center	48(56.5)	16(55.2)	32(57.1)	0.862
Corpus callosum	27(31.8)	6(20.7)	21(37.5)	0.115
Thalamus	16(18.8)	5(17.2)	11(19.6)	0.788
Cerebellum	27(31.8)	7(24.1)	20(35.7)	0.277
Medulla oblongata	33(38.8)	11(37.9)	22(39.3)	0.903
Pons	47(55.3)	14(48.3)	33(58.9)	0.349
Midbrain	13(15.3)	3(10.3)	10(17.9)	0.552
Patients with spinal cord MRI abnormalities (n, %) **	64(77.1)	30(100.0)	34(64.2)	<0.001
Cervical	57(70.4)	29(96.7)	28(54.9)	<0.001
Thoracic	41(64.1)	20(74.1)	21(56.8)	0.154
≥3 spinal lesions	47(57.3)	24(80.0)	23(44.2)	0.002
Isolated cervical	24(29.3)	11(36.7)	13(25.0)	0.263
Isolated thoracic lesions	8(9.8)	2(6.7)	6(11.5)	0.742
Cervical and thoracic	31(37.8)	16(53.3)	15(28.8)	0.028
Upper thoracic lesions	31(37.8)	15(50.0)	16(30.8)	0.084
Lower thoracic lesions	29(35.4)	14(46.7)	15(28.8)	0.104
≥4 thoracic lesions	17(21.0)	8(26.7)	9(17.6)	0.336
Pain locations				
Head	18(20.5)			
Neck/shoulder	14(15.9)			
Upper limb	18(20.5)			
Thorax	5(5.7)			
Abdomen	6(6.8)			
Back	15(17.0)			
Lower limb	22(25.0)			

EDSS, Extended disability status Scale, Upper thoracic lesions=Th1-Th6, lower thoracic lesions=Th7-Th12.

*Missing 3 cases.

**Missing 6 cases.

The total prevalence of neuropathic pain in our study was 34.1% (30/88). The prevalence of ongoing extremity pain, trigeminal neuralgia and Lhermitte’s phenomenon was separately 21.6%, 3.4% and 4.5%. No significant difference was observed between NP and non-NP group in terms of gender (P = 0.575), age of onset (P = 0.262) and EDSS (P = 0.055). A higher number of cases with extended spinal cord lesions (80.0% vs. 44.2%, P = 0.002), cervical and thoracic lesions (53.3% vs. 28.8%, P = 0.028), were observed in NP group. No significant difference was observed between the groups in relation to upper and lower thoracic lesions (p > 0.05). In addition, non-NP group had higher basal ganglia lesions (20.7% vs. 48.2%; P = 0.014) and temporal lobe lesions (20.7% vs. 42.9%; P = 0.043) compared to NP group (**Table 1**).

The multivariate logistic regression model found that extended spinal lesions (≥3 spinal lesions) (OR 11.878, 95% CI 2.930-48.157, P = 0.001), cervical and thoracic lesions (OR 4.276, 95% CI 1.366-13.382, P = 0.013) and upper thoracic lesions (T1-T6) (OR 3.047, 95% CI 1.018-9.124, P = 0.046) were identified as risk factors for neuropathic pain among MS patients. By contrast, those with basal ganglia lesions (OR 0.188, 95% CI 0.044-0.809, P = 0.025) had lower risk. Furthermore, we categorized the patients according to the tertiles of the spinal lesions (0-1, 2-4, >5) and the results indicated that the longer the spinal lesions, the greater the neuropathic pain risks (2-4: OR, 13.3(2.1-82), >5: OR, 15.2(2.7-86.8), p for tread: 0.037). Meanwhile, age might be associated with neuropathic pain (OR 1.046, 95% CI 1.000-1.094, P=0.051), too (**Table 2**, **3**).

**Table 2:** The multivariate analysis of risk factors of neuropathic pain.

	Crude OR (95% CI)	P value	Adjusted model OR (95% CI)	P value
Age	1.019(0.987-1.052)	0.260	1.046(1.000-1.094)	0.051
Gender, female	1.312(0.506- 3.403)	0.576	1.367(0.370-5.059)	0.639
EDSS	1.276(1.026-1.588)	0.029	1.230(0.939-1.613)	0.133
Frontal lobe ^a^	0.458(0.183-1.148)	0.096	1.545(0.368-6.484)	0.552
Temporal lobe ^a^	0.348(0.123-0.987)	0.047	0.474(0.110-2.039)	0.316
Basal ganglia ^a^	0.280(0.099-0.793)	0.017	0.188(0.044-0.809)	0.025
≥3 spinal lesions ^b^	5.043(1.767-14.394)	0.002	11.878(2.930-48.157) *	0.001
Cervical and thoracic lesions ^b^	2.819(1.107-7.181)	0.030	4.276(1.366-13.382) *	0.013
Upper thoracic lesions ^b^	2.250(0.891-5.685)	0.086	3.047(1.018-9.124) *	0.046

Adjusted for age, gender, EDSS, frontal lobe, temporal lobe, basal ganglia, ≥3 spinal lesions, cervical and thoracic lesions, upper thoracic lesions.

* Adjusted for age, gender, EDSS, frontal lobe, temporal lobe, basal ganglia.

a Missing 3 cases.

b Missing 6 cases.

**Table 3:** Multivariate regression analysis.

Model	Number of spinal lesions	OR (95% CI)	P	P for trend
1	0-1	Ref		0.037
	2-4	4.4(1.2-17.0)	0.030	
	>5	5.7(1.6-20.4)	0.007	
2	0-1	Ref		
	2-4	6.7(1.5-29.9)	0.013	
	>5	8.2(2.0-34.5)	0.004	
3	0-1	Ref		
	2-4	13.3(2.1-82.0)	0.005	
	>5	15.2(2.7-86.8)	0.002	

*Missing 6 cases.

Model 1: crude model.

Model 2: adjusted for age, gender and EDSS.

Model 3: adjusted for age, gender, EDSS, frontal lobe, temporal lobe, basal ganglia.

## 4 Discussion

Our study identified a significant association between the spinal cord lesions and neuropathic pain among MS patients.

### 4.1 The criteria and prevalence of neuropathic pain of MS

Neuropathic pain is a common and serious symptom of MS that greatly affects the patients’ quality of life. According to previous studies, the diagnosis of neuropathic pain was mainly defined through the following methods: IASP criteria (15) AAPT criteria (19), questionnaire evaluation (20), and clinical symptoms (21). Douleur Neuropathique 4 Questions (DN4), McGill Pain Questionnaire (MPQ) and Pain Disability Questionnaire (PDQ), are the most commonly used for the diagnosis of neuropathic pain(11). In Heitmann’s study, neuropathic pain was defined as PDQ ≧̸19 and the prevalence of neuropathic pain was 4.2% (20). Solaro’s study used DN4 scale combining with the criteria of the IASP to diagnose the neuropathic pain and the incidence of neuropathic pain is 13.8% (15). Therefore, there were different criteria of neuropathic pain and the prevalence of neuropathic pain varied in different studies.

In our study, we also found the prevalence of the headache was 20.5%. According to the literature, headache is common in the population with MS. The prevalence of headache in MS patients was 2-56% (22, 23). However, the mechanism and influence of headache on MS are still unclear (24). More research is needed for exploration in the future.

### 4.2 Extended spinal lesions were independent risk factors of neuropathic pain

Consistent with previous studies, 34.1% of patients experienced neuropathic pain in our study. Meanwhile, we found that extended spinal lesions (≥3 spinal lesions), cervical and thoracic lesions and upper thoracic lesions (T1-T6) independently contributed to the risk of neuropathic pain among patients with MS. The spinal cord plays a crucial role in the pathogenesis of pain, especially in neuromyelitis optica spectrum disorder (NMOSD) and MS. As is well known, neuropathic pain of MS is caused by demyelinating lesions in pain perception areas (brain and spinal cord). The spinothalamic tract is the main sensory ascending pathway, consisting of the anterior spinothalamic tract that transmits tactile sensation and the lateral spinothalamic tract that transmits pain and warmth sensation. Previous study had indicated that spinothalamic dysfunctions was the mechanism of MS patients with neuropathic pain (12, 25). Therefore, damage to the spinothalamic tract may be a possible mechanism for the occurrence of neuropathic pain in patients with spinal cord lesions. Another suspected mechanism might be related to the destruction of the autonomic intermediomedial nucleus located in the upper/middle segment of the thoracic spinal cord. These neurons project bilaterally to the superficial dorsal horn of the lumbosacral region, which may contribute to the occurrence of pain (26). In addition, from anatomical and pathophysiological perspective, lesions in the thalamus and parietal cortex are likely related to neuropathic pain and these association were verified by some studies (27, 28). However, in our study, we didn’t find the association of parietal lobe lesions as well as the thalamic lesions with the neuropathic pain which should be investigated by further studies.

Although spinal cord lesions were of vital importance in the diagnosis and monitoring of disease recurrence and progression of MS, few studies have investigated the relation between pain and the length of involved spinal segments in patients with MS. The association between spinal cord and neuropathic pain is still controversial. Research had showed that MS patients with upper/mid-thoracic spinal cord demyelinating lesions were more likely to experience neuropathic pain (OR 155.0, 95% CI 17.0–1414.0, P < 0.001) (26). Similarly, some studies also showed that thoracic cord lesions, especially the upper thoracic lesions (T1-T6) were closely related to pain among NMOSD patients, and indicated more severe pain (29–31). Likewise, our previous study has shown that extended thoracic lesions (≥4 thoracic lesions) were strong predictors of neuropathic pain in NMOSD patients (18). On the basis of this study, our further exploration found cervical and thoracic lesions and upper thoracic lesions (T1-T6) are positively associated with neuropathic pain.

Furthermore, we found that the longer the spinal lesions, the greater the neuropathic pain risks (2-4: OR 13.3(2.1-82), >5, OR 15.2(2.7-86.8), p for tread: 0.037). This is the first report to demonstrate the linear correlation between the length of spinal cord lesions and neuropathic pain in MS. Our study found association between neuropathic pain and spinal cord lesions levels in MS, similar in some respects to NMOSD. These results suggested that NMOSD and MS may develop with common mechanisms in patients with neuropathic pain. Because the exploration and research on the pain mechanism and risk factors of MS were scare, more prospective cohort studies were needed.

### 4.3 Basal ganglia lesions might not be risk factors of neuropathic pain

In our study, basal ganglia lesions (OR 0.188, 95% CI 0.044-0.809, P = 0.025) were negatively correlated with neuropathic pain in MS patients. The basal ganglia was considered as a brain region of nociceptive sensorimotor integration (32). Painful diseases of the nervous system such as Parkinson (33), migraine (34) and Huntington’s disease (35), have been proposed to be related to the basal ganglia. In Parkinson’s disease, deep brain stimulation (DBS) may alleviate pain symptoms, with the subthalamic nucleus (STN) located in the basal ganglia circuit as the most commonly used target for treatment. Research has shown that in Parkinson’s disease, abnormal and pathological enhancement of STN activity was observed, and pain sensitivity and central sensitization in Parkinson’s disease mice could be improved by inhibiting overactive STN neurons. The underlying mechanism may relate to a pathway which is composed of STN, the substantia nigra pars reticulata and the lateral parabrachial nucleus (36). The destructed inhibitory inputs of the pathways might lead to inadequate response to pain, which may explain the negative correlation between basal ganglia lesions and neuropathic pain in our cohort. However, other studies of MS didn’t find such relationship between basal ganglia lesions and neuropathic pain. Further studied were needed to explore the possible association.

### 4.4 Gender and age may not associate with neuropathic pain

The correlation between gender, age and neuropathic pain in patients with MS was inconsistent in previous studies. Recently, a meta-analysis surveyed 6671 MS patients and found that prevalence of neuropathic pain was higher for females (74.17%, 95% CI: 65.34; 81.38) than male patients (28.93%, 95% CI: 22.28; 36.63) (11). Meanwhile, the possible mechanisms that contribute to this phenomenon might be the influence of sex hormones, genetic and anatomical differences (37). However, another study found neuropathic pain in MS patients is not correlated with gender (females vs males = 27.5% vs 36.4%, p = 0.715) (38). As to the association between age and neuropathic pain, some suggested that they had no association (39, 40). Conversely, another previous study reported that the presence of neuropathic pain is associated with age [OR 1.03, 95% CI 1.01–1.04, P = 0.002] in MSF (15). In our study, we suggested that neuropathic pain is neither associated with gender nor with age. However, we found a trend towards increasing neuropathic pain alone with age (OR 1.046, 95% CI 1.000-1.094, P = 0.051). Larger studies were need to confirm the association of neuropathic pain and demographic features.

### 4.5 Limitation

There were some limitations of the present study. Firstly, the evaluation of the scales was not obtained while the retrospective data collection. Therefore, we cannot determine whether intracranial and spinal cord lesions were associated with the severity and pain-related accompanying symptoms of neuropathic pain. Secondly, results from single-center study are less responsive to broader population, larger cohort studies were need to explore in more detail.

## 5 Conclusions

Our study indicated that for MS patients, the longer the spinal lesions, the greater the neuropathic pain risks. Meanwhile, extended spinal lesions (≥3 spinal lesions), cervical and thoracic lesions, upper thoracic lesions (T1-T6) were independent risk factors of neuropathic pain. Early attention to spinal cord lesions may be beneficial for pain management in MS patients.

## Data availability statement

The raw data supporting the conclusions of this article will be made available by the authors, without undue reservation.

## Ethics statement

The studies involving humans were approved by Ethical Committee of Guangdong Provincial Hospital of Chinese Medicine. The studies were conducted in accordance with the local legislation and institutional requirements. The ethics committee/institutional review board waived the requirement of written informed consent for participation from the participants or the participants’ legal guardians/next of kin because data were collected retrospectively and analyzed anonymously, individual patient consents were not required.

## Author contributions

HO: Conceptualization, Data curation, Formal analysis, Investigation, Software, Visualization, Writing – original draft. XL: Data curation, Formal analysis, Investigation, Validation, Writing – review & editing. HX: Resources, Validation, Writing – review & editing. YiZ: Data curation, Investigation, Writing – review & editing. ZZ: Resources, Validation, Writing – review & editing. GC: Data curation, Writing – review & editing. ZL: Data curation, Writing – review & editing. HC: Data curation, Writing – review & editing. JZ: Data curation, Writing – review & editing. HM: Data curation, Writing – review & editing. CZ: Data curation, Writing – review & editing. LQ: Data curation, Writing – review & editing. YuZ: Funding acquisition, Project administration, Supervision, Writing – review & editing. MZ: Methodology, Project administration, Software, Supervision, Writing – review & editing.
